# Diversity of family GH46 chitosanases in *Kitasatospora setae* KM-6054

**DOI:** 10.1007/s00253-017-8517-9

**Published:** 2017-09-18

**Authors:** Mina Zitouni, Pascal Viens, Mariana G. Ghinet, Ryszard Brzezinski

**Affiliations:** 1 Centre d’Étude et de Valorisation de la Diversité Microbienne; Département de Biologie; Faculté des Sciences, Université de Sherbrooke, 2500, boul.de l’Université, Sherbrooke, Québec J1K 2R1 Canada; 2 Innomalt Inc, Sherbrooke, Québec Canada; 3 Present Address: Département de Pharmacologie, Faculté de Médecine et des Sciences de la Santé, Université de Sherbrooke, Sherbrooke, Québec Canada

**Keywords:** Chitosan, Chitosanase, *Kitasatospora*, Actinomycetes, Glycoside hydrolase, Genome mining

## Abstract

The genome of *Kitasatospora setae* KM-6054, a soil actinomycete, has three genes encoding chitosanases belonging to GH46 family. The genes (*csn1*-*3*) were cloned in *Streptomyces lividans* and the corresponding enzymes were purified from the recombinant cultures. The *csn2* clone yielded two proteins (Csn2BH and Csn2H) differing by the presence of a carbohydrate-binding domain. Sequence analysis showed that Csn1 and Csn2H were canonical GH46 chitosanases, while Csn3 resembled chitosanases from bacilli. The activity of the four chitosanases was tested in a variety of conditions and on diverse chitosan forms, including highly *N*-deacetylated chitosan or chitosan complexed with humic or polyphosphoric acid. Kinetic parameters were also determined. These tests unveiled the biochemical diversity among these chitosanases and the peculiarity of Csn3 compared with the other three enzymes. The observed biochemical diversity is discussed based on structural 3D models and sequence alignment. This is a first study of all the GH46 chitosanases produced by a single microbial strain.

## Introduction

Chitosan is a polymeric amino sugar composed of a majority of D-glucosamine (GlcN) units and a minority of *N*-acetyl-d-glucosamine units (GlcNAc) united by β-1,4-glycosidic links. The degree of *N*-deacetylation (DDA), an important parameter determining the chemical and biological behavior of chitosan, is defined as the proportion of GlcN groups in a given sample of chitosan (Roberts 1992). Chitosan is present in the cell walls of several groups of microorganisms, like *Zygomycetes* fungi or microalgae (Bartnicki-Garcia 1968, Kapaun and Reisser 1995). Starting from raw materials containing chitin, such as crustacean shells (essentially shrimps and crabs), chitosan is produced at an industrial scale by alkaline treatment. Chitosan has numerous potential applications in medicine and technology, which justifies its production at the level of hundreds of tons per year (Hamed et al. 2016).

Chitosan is chemically more reactive than most polysaccharides due to the free amino groups, which are deprotonated at mildly acidic pH (the isoelectric point of chitosan is around 6.2). Considering the complexity of the constituents of natural environments, chitosan could be present in a wide variety of forms: more or less *N*-deacetylated, in a protonated or deprotonated form (depending on surrounding pH), forming salts with environmental acids, chelating metals, or complexed with polymers. However, chitosan was rarely considered in ecological studies.

Chitosan can be hydrolyzed by endo-hydrolases as chitosanases, chitinases, or lysozymes which activity often depends on the DDA of chitosan (Hoell et al. 2010). The hydrolysis, initiated by these endo-acting enzymes and resulting in dimeric or longer products, can be completed by exo-acting enzymes like glucosaminidases and *N*-acetyl-glucosaminidases, generating, respectively, GlcN and GlnNAc monomers. All these enzymes are widely represented in living organisms (Hoell et al. 2010).

Chitosans with moderate or high DDA values are preferentially hydrolyzed by chitosanases. The best characterized chitosanases belong to families GH46, GH8, and GH80 of glycoside hydrolases and their catalytic mechanisms as well as structure-function relationships were described in great detail, essentially due to crystallographic and site-directed mutagenesis studies (Adachi et al. 2004, Liu et al. 2015, Viens et al. 2015b, Yorinaga et al. 2017). However, almost all chitosanase studies have been dedicated to only one enzyme per microbial strain. Multiple chitosanases originating from a single microbial strain were rarely reported. Therefore, it remains largely unknown how a single bacterial species deals with the wide variety of chitosan forms potentially present in nature.

Pelletier and Sygusch (1990) isolated a strain of *Bacillus megaterium* from soil and characterized three chitosanases (named A, B, and C) produced by this strain. All were acting as endo-hydrolases. They differed by their relative abundance in culture supernatant, their dependence of their activity on DDA of chitosan substrate, their specific activity against high-DDA chitosan, and their capacity to hydrolyze cellulosic substrates. While limited to biochemical aspects, this study revealed for the first time that a single bacterial species could produce several chitosanases, each with different properties.

In *Aspergillus oryzae* IAM2660, a mold used in the production of fermented food in Japan, three chitosanase genes (*csnA*, *B*, and *C*) belonging to GH75 family have been cloned and sequenced but only the chitosanase CsnC was characterized in detail (Zhang et al. 2000; Sugita et al. 2012). This chitosanase is atypical as it includes three tandem-repeated peptides forming the so-called R3 domain at the C-terminus. This domain had the ability to bind to insoluble chitosan but did not influence significantly the enzyme activity.

Three genes encoding putative GH46 chitosanases have been detected in the genomic sequence of *Streptomyces* sp. SirexAA-E (Takasuka et al. 2014). Only one chitosanase, SACTE_5457 has been studied in detail (including 3D-structure determination following crystallization) as the proteins encoded by the other two genes could not be detected in any of the tested culture conditions. Finally, two chitosanases were detected in the chitosan-assimilating strain *Acinetobacter* sp. CHB101 (Shimosaka et al. 1995). These enzymes differed essentially by their substrate preference, chitosanase II being able to hydrolyze efficiently glycol chitin as well as 70%-deacetylated chitosan, while chitosanase I preferred highly deacetylated chitosan.

Exploring the carbohydrate active enzymes database (www.cazy.org) (Lombard et al. 2014), we identified *Kitasatospora setae* KM-6054 as one strain having three genes encoding putative chitosanases belonging to GH46 family. We present in this work for the first time an exhaustive comparative study of all the GH46 chitosanases encoded by a single strain; furthermore, we compare two forms of the same enzyme encoded by one of the chitosanase genes. Finally, we discuss the molecular basis of the observed biochemical diversity.

## Materials and methods

### Bacterial strains

*Kitasatospora setae* KM-6054 (Ōmura et al. 1981) was obtained from the microbial collection of the Kitasato University, Tokyo, Japan (also available as ATCC 33774). *Escherichia coli* DH5α (Hanahan 1985) was used for plasmid propagation. *Streptomyces lividans* TK24 ∆*csnR* (formerly *∆2657* h) (Dubeau et al. 2011a; Brzezinski 2011) was used as host for recombinant plasmids directing chitosanase production.

### Media and culture conditions

Luria-Bertani (broth or agar) medium was used for all manipulations involving *E. coli* (Hanahan 1985). Routine propagation of *K*. *setae* was done on yeast-malt extract (YME) medium (glucose, 4 g L^−1^; yeast extract, 4 g L^−1^; malt extract, 10 g L^−1^).

Native and recombinant *S*. *lividans* TK24 ∆*csnR* strains were grown on tryptic soy broth (TSB) or tryptic soy agar (TSA). Spores of *S*. *lividans* were collected after heavy inoculation of oatmeal agar plates followed by 1 week of incubation. Incubation of *E. coli* or actinobacterial strains on all media was at 37 or 30 °C, respectively. All media components were from Difco Laboratories.

### Construction of recombinant plasmids

All the genetic constructions were performed using the shuttle vector pFDES, a derivative of pFD666 obtained after deleting a segment delimited by unique *Nru*I and *Acl*I sites (Sanssouci et al. 2011). All plasmids have been constructed and propagated using *E. coli*, and the final constructions were transformed into *S*. *lividans* TK24 ∆*csnR*. The genes were amplified from genomic DNA by PCR using primers listed in Table 1. Amplicons for *csn1* and *csn2* genes were digested with *Hin*dIII and *Sca*I and ligated to pFDES vector digested with the same restriction enzymes. For *csn3*, a synthetic DNA segment (containing the transcription promoter and the ribosome-binding site) digested with *Hin*dIII and *Nde*I was ligated with the *csn3*-amplicon digested with *Nde*I and *Bam*HI and the pFDES vector digested with *Hin*dIII and *Bam*HI. Synthetic DNA (Table 1) was obtained from Biomatik (Cambridge, Ontario, Canada). Amplification of gene segments was performed with Q5 Hot Start High Fidelity polymerase (New England BioLabs, Ipswich, MA, USA) after primer/DNA annealing at 69 °C (*csn1* and *csn3*) or 66 °C (*csn2*). DNA primers were obtained from Integrated DNA Technologies (Coralville, IA, USA).

**Table 1:** Primers and synthetic DNA used for chitosanase gene cloning

Name	Sequence (5′−3′)	Coordinates of homologous genomic segment covered by the primer	Length of amplified genomic segment
Forward Csn1	NNNNNNNNNNAAGCTTCCGGATGCCGTCAGACGT	1,679,152–1,679,135	1101 bp
Reverse Csn1	NNNNNNNNNNAGTACTGTCCGTCAGCGAAGCGAA	1,678,052–1,678,069	
Forward Csn2	NNNNNNNNNNAAGCTTCCTGATGGTCGGTCATC	4,542,402–4,542,421	1586 bp
Reverse Csn2	NNNNNNNNNNAGTACTGCGGAGACCCGTTCGTTA	4,543,987–4,543,970	
Forward csn3	TTCGCAAGGAGAACCATATGCGC	4,551,090–4,551,112	988 bp
Reverse csn3	NNNNNNNNNNGGATCCACCACTCCGTGCAATGGAAC	4,552,052–4,552,032	
Synthetic DNA (promoter and RBS for csn3)	AAGCTTGAATTCAATTGCCCAC**TTGACG**TTGAGAGTGAAGCAATA**TAGGTT**AACCTCGGTTCGAAACCAGGAGACGTACATATG		

Restriction sites used for cloning are underlined. Bold characters in the synthetic DNA sequence indicate the − 35 and − 10 boxes of the promoter sequence D1-7 as identified by Seghezzi et al. (2011)

### Chitosanase production and purification

Spores of *S*. *lividans ∆csnR* harboring the recombinant plasmids were inoculated into TSB medium supplemented with 50 μg mL^−1^ kanamycin, and the cultures were incubated for 48 h at 30 °C with shaking (230 rpm min^−1^). Mycelium was recovered by low-speed centrifugation and inoculated in a modified M14 medium (Pagé et al. 1996): composed of KH_2_PO_4_, 1 g L^−1^; K_2_HPO_4_, 5 g L^−1^; NH_4_Cl, 1 g L^−1^; K_2_SO_4_, 1 g L^−1^; and 1 ml L^−1^ of trace elements solution (CoCl_2_·7H_2_O, 2 mg mL^−1^; FeSO_4_·7H_2_O, 5 mg mL^−1^; MnSO_4_·H_2_O, 1.6 mg mL^−1^, and ZnSO_4_·7H_2_O, 1.4 mg mL^−1^), pH 6.9. After autoclaving, MgSO_4_, 0.3 g L^−1^ and CaCl_2_, 0.3 g L^−1^ were added to the M14 medium. The resulting mineral solution was supplemented with 10 g L^−1^ mannitol as carbon source. Cultures were incubated at 30 °C with shaking (230 rpm min^−1^). Incubation time was 48 h for Csn1 and Csn2 and 70 h for Csn3. For enzyme purification, culture supernatants were recovered after low-speed centrifugation (20 min, 4000*g* at 4 °C).

Csn1 was purified using a procedure previously published for chitosanase from *Streptomyces* sp. N174 without modifications (Lacombe-Harvey et al. 2009).

For Csn2 isoforms (Csn2BH and Csn2H) purification, the culture supernatant was adjusted to pH 7.5 with Tris base, then diluted with distilled water to attain a conductivity of 4 mS cm^−1^ and loaded on a Q-XL Sepharose column (GE Healthcare) equilibrated with 20 mM Tris-HCl buffer pH 7.5. Elution was performed with 0–0.6 M NaCl gradient in the same buffer. Active fractions were pooled, dialyzed against unbuffered 1 mM MgCl_2_, and loaded on Hydroxyapatite Fast Flow (Calbiochem) column (8 × 1.6 cm) pre-equilibrated with unbuffered 1 mM MgCl_2_. Elution was performed with five column volumes unbuffered 1 mM–1 M MgCl_2_ gradient at 2 mL min^−1^. At this stage, both Csn2 forms were coeluted. To separate Csn2BH from Csn2H, chitosanase active fractions were pooled, dialyzed against 20 mM Tris-HCl + 150 mM NaCl, and then loaded onto a size exclusion Sephacryl S-100 HR column (80 × 1.6 cm) equilibrated with the same buffer and eluted under gravity.

For Csn3 purification, the culture supernatant was adjusted to pH 8 with Tris base, then diluted with distilled water to attain a conductivity of 4 mS cm^−1^ and loaded on a Q-XL Sepharose column equilibrated with 20 mM Tris-HCl pH 8. Elution was performed with 0–0.6 M NaCl gradient in the same buffer. Active fractions were pooled, dialyzed against unbuffered 1 mM MgCl_2_, and loaded on Hydroxyapatite Fast Flow column (5 × 1.6 cm) pre-equilibrated with unbuffered 1 mM MgCl_2_. The column was washed with unbuffered 1 M MgCl_2_, then with 10 mM potassium phosphate pH 6.8. Elution was performed with a 10–300 mM potassium phosphate buffer pH 6.8 gradient.

All four purified enzyme preparations were dialyzed against 50 mM Na-acetate buffer pH 5.5 and stored at 4 °C. They were supplemented with 50% glycerol for long-term storage at − 20 °C.

### Biochemical assays

The purity of the enzyme preparations was evaluated by SDS-PAGE (Laemmli 1970) using the PageRuler Protein Ladder (Thermo Fisher Scientific, Waltham, MA, USA) as molecular weight standard. Protein concentration was determined by the method of Bradford (1976) using bovine serum albumin as standard (Sigma). Protein concentration of purified chitosanases was determined by ultraviolet absorbance (280 nm) using the following molar extinction coefficients (in M^−1^ cm^−1^): 32890 for Csn1, 76320 for Csn2BH, 35410 for Csn2H, and 25440 for Csn3. The coefficients were estimated from amino acid sequences using the ProtParam tool on ExPASy server (Gasteiger et al. 2005) following the rules established by Gill and von Hippel (1989). To determine the exact N-termini for Csn2BH and Csn2H, a proteomic analysis was performed by Plateforme Protéomique (Centre de Génomique de Québec, Université Laval, Québec, QC, Canada).

Chitosanase activity was assayed by two methods. Rapid assay in culture supernatants and fractions eluted from chromatographic columns was performed using the soluble chitosan derivative sRBB-C as substrate (Zitouni et al. 2010). For all other experiments, chitosanase activity was measured by the release of reducing sugars from chitosan substrate with d-glucosamine as standard by the method of Lever (1972), using the *p*-hydroxybenzoic acid (PAHBAH) reagent as modified by Schep et al. (1984). Readings of optical density at 405 nm were performed on 200 μl aliquots of reaction supernatant in 96 well plates using an Asys UVM 340 microplate reader (UK Biochrom). One unit of chitosanase was defined as the enzyme quantity which releases 1 μmol of d-glucosamine equivalent per min from standard chitosan substrate in a 10-min reaction at 37 °C.

The standard substrate solution was prepared with commercial chitosan obtained from Sigma-Aldrich, dissolved at 10 g L^−1^ in 0.2 M acetic acid (stock solution), and diluted to a final concentration of 0.5 mg mL^−1^ in 50 mM Na-acetate buffer pH 5.5. For kinetic experiments, the same stock solution was diluted toward concentrations ranging from 0.01 to 0.15 mg mL^−1^. To test the dependence of activity on the degree of deacetylation of substrate, solutions of chitosans deacetylated at 84.6 and 98.4% (Marinard, Québec) were prepared in the same way.

For the tests at low temperatures or in the presence of humic acids or polyphosphoric acid, all the chitosanase samples were first diluted in 50 mM Na-acetate buffer pH 5.5 in order to give approximatively the same release of reducing sugars in a standard assay for 10 min at 37 °C with standard chitosan substrate. This activity was taken as a reference (100%) for comparison with the other conditions tested. For tests at low temperatures, the aliquoted standard substrate solution (480 μL) was equilibrated at a given temperature before the addition of enzyme solution (20 μL). Reactions were incubated for 20 min (for temperatures of 30, 25, 20, and 15 °C) or 30 min (for temperatures of 10 and 7 °C) and stopped by the addition of 500 μL of PAHBAH reagent.

The polyphosphoric acid-chitosan substrate was prepared as follows. Polyphosphoric acid liquid concentrate (Sigma-Aldrich C3646) was diluted up to 1 mg mL^−1^ in Na-acetate buffer (pH 4.5 or 5.5). The chitosan component was prepared in the same buffer at 1 mg mL^−1^. To prepare 480 μL of substrate, 120 μL of buffered polyphosphoric acid solution was combined with 240 μL of chitosan solution and completed with 120 μL of Na-acetate buffer at the appropriate pH. The combined substrates were kept overnight at room temperature and then incubated for 10 min at 37 °C before addition of the enzyme. Reaction time was 100 min.

For tests of activity in the presence of humic acids, the humic acid powder (Agros Organics AC120861000) was combined with 50 mM Na-acetate buffer (pH 4.5 or 5.5) to the final concentration of 2 mg mL^−1^. The chitosan solution was also prepared at a concentration of 2 mg mL^−1^ in the same buffer. To prepare 480 μL of substrate, 185 μL of humic acid solution was combined with 125 μL of chitosan solution and completed with Na-acetate buffer of appropriate pH. The combined substrates were kept overnight at room temperature and then incubated for 10 min at 37 °C before addition of the enzyme. Reaction time was 10 min at pH 4.5 and 15 min at pH 5.5.

Undissolved (suspended) chitosan substrate was prepared by adding 50 mg of chitosan from Sigma-Aldrich to 10 ml of 50 mM Na-acetate buffer pH 5.5. The suspension was mixed by vortexing then kept 30 min at room temperature. Chitosan was recovered by centrifugation (20 min at 4000*g* then the pellet was suspended in 50 ml of the same buffer, and vortexed and used immediately for reaction with enzyme (30 min at 37 °C in a rotary agitator).

All reactions with chitosan/acid mixtures and undissolved chitosan were done in six independent replicas. Results were analyzed by the statistical Tukey’s multiple comparisons test using GraphPad Prism software version 7.01.

## Results

### *Kitasatospora setae* genome mining and rationale of gene cloning

**Fig. 1:**
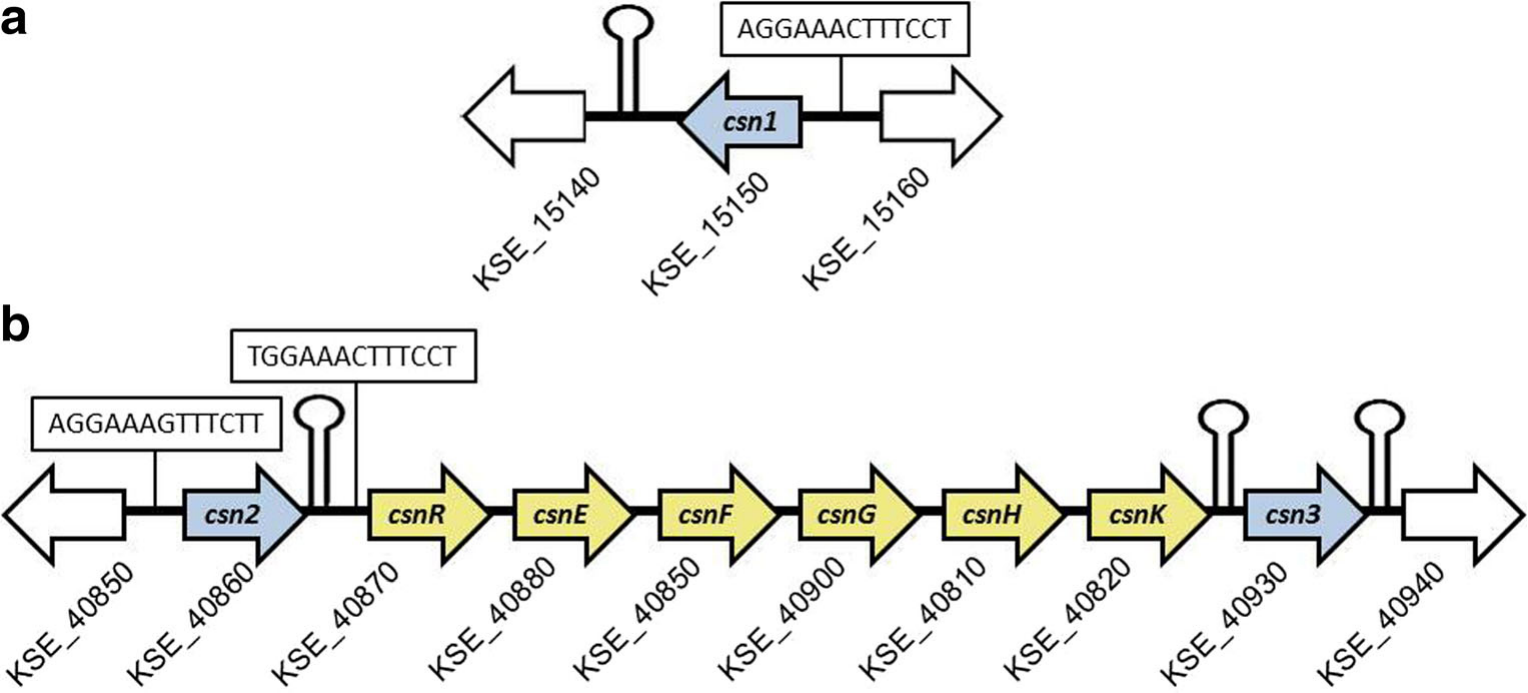
Arrangement of GH46 chitosanase genes in the genome of *K*. *setae*. Gray arrows: chitosanase genes. Yellow arrows: genes belonging to a putative glucosamine oligosaccharide transport operon (Viens et al. 2015a). White arrows: flanking genes. The numbers of the genes from GenBank sequence file are also indicated. *csnR*: chitosanase gene repressor; *csnEFG*: ABC transporter; *csnH*: GH4 family glycoside hydrolase; and *csnK*: putative oligosaccharide kinase. Boxed sequences: putative operators recognized by CsnR repressor: stem and loops: putative palindromic terminators of transcription

The CAZy database reveals three GH46 family genes in the genome of *K*. *setae* KM-6054; the first *Kitasatospora* genome to be entirely sequenced (Ichikawa et al. 2010). The *csn1* gene (= *KSE*_*15150*) encodes a chitosanase (Csn1) whose primary sequence is rather similar to other well studied enzymes such as the chitosanases CsnN174 from *Streptomyces* sp. N174 and OU01 from *Microbacterium* sp., as illustrated by the alignments and trees presented by Takasuka et al. (2014) and Viens et al. (2015b). The *csn1* gene forms most probably a monocistronic transcription unit, being flanked by a putative transcription terminator and a gene transcribed in an opposite direction (Fig. 1a).

Interesting features were found for the other two GH46 genes/proteins (Fig. 1b). The *csn2* and *csn3* genes are localized on both sides of a six-gene cluster led by an ortholog of *csnR*, which we described previously (Dubeau et al. 2011b; Viens et al. 2015a). While this gene cluster, dedicated to the regulation of chitosanase gene expression and the transport of GlcN oligosaccharides resulting from chitosanase hydrolysis, is highly conserved in actinobacterial genomes, this is the first reported case where this cluster is accompanied by flanking chitosanase genes.

The chitosanases encoded by these genes also proved to be unusual among GH46 members. The Csn2 is distinctive because of the presence of a carbohydrate-binding module (CBM) between the signal peptide and the hydrolytic module, which is rare in GH46 family. Identified in protein databases as the F5_F8_type_C domain (pfam00754) or CBM32, the CBM present in Csn2 is highly similar to two domains, DD1 and DD2 (46 and 45% of identity; 63 and 61% of similarity, respectively), present in the GH8 chitosanase from *Paenibacillus* sp. IK-5 and shown to function as binding modules specific to chitosan (Shinya et al. 2013). Otherwise, the hydrolytic module of Csn2 is similar to Csn1 and CsnN174 (Viens et al. 2015b).

Chitosanase Csn3 belongs to group B inside GH46 family, as defined by Viens et al. (2015b). Members of this group are frequent in bacilli but rare in actinobacteria and, according to the CAZy database, *K*. *setae* in one of only a few actinobacteria in which such a chitosanase is present.

To determine the cloning strategy, we analyzed upstream and downstream sequences from each ORF. When found, putative downstream transcription terminators of the “stem and loop type” (Fig. 1) were included in the segments amplified by PCR from genomic DNA. Putative transcription promoters were found in the upstream segments of *csn1* and *csn2* genes and were also included in the amplicons. As a consequence, these two amplified segments also included palindromic sequences localized between the putative promoter and the ribosome-binding site, and representing, most probably, the operators for binding of the repressor CsnR (Fig. 1), suggesting that these two genes were negatively regulated in a mode analogous to the one described for *S*. *lividans* (Dubeau et al. 2011a, b). As these operators were almost identical with those found in *S*. *lividans*, the negative regulation could be kept in this heterologous host, making the expression of *csn1* and *csn2* dependent on the presence of chitosan in the medium. To avoid this, the recombinant genes were transformed into an *S*. *lividans* host with deleted *csnR* gene (Dubeau et al. 2011a, Brzezinski 2011). In the case of *csn3*, we could not identify any sequence similar to a known actinobacterial promoter in the upstream segment. Thus, in the recombinant construction, a synthetic DNA segment including the strong promoter D1-7 (Seghezzi et al. 2011) and a ribosome-binding site was ligated to the amplified ORF (Table 1), providing the necessary elements for initiation of transcription and translation.

### Protein production and purification

**Fig. 2:**
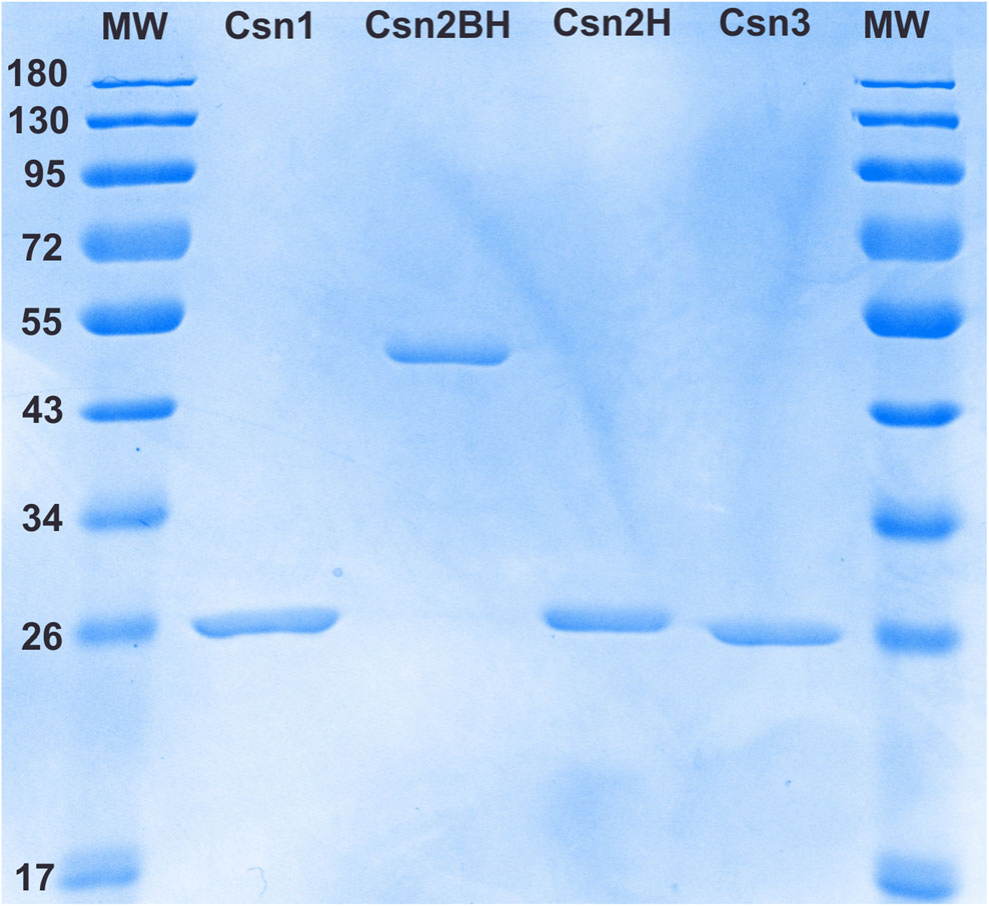
SDS-PAGE analysis of purified chitosanases. MW molecular weight marker

For chitosanase production, the recombinant strains were cultivated in a defined medium, containing only inorganic salts and mannitol as carbon source. Chitosanases were purified to apparent homogeneity (Fig. 2). During the purification of Csn2, it became apparent that the enzyme was present in two forms differing by their molecular weight. Both forms had largely coinciding peaks in the early steps of purification but could be separated by size-exclusion chromatography. Proteomic analysis was undertaken to confirm the identity of both purified polypeptides. It showed that the high-molecular weight polypeptide represented the full-length mature protein, containing both the carbohydrate-binding module at N-terminus and the hydrolytic module at the C-terminus, while the low-molecular weight polypeptide included only the hydrolytic module. The polypeptides were named Csn2BH and Csn2H, respectively. We also learned that Csn2H was generated from Csn2BH by cleavage in a Ser-Ala-Pro-rich segment, more precisely at the following site: TASASASA↓SPTASPSP.

The following specific activities (in units per milligram of protein) were determined for the purified enzymes by standard assay with chitosan Sigma: 19.2 for Csn1, 12.6 for Csn2BH, 20.1 for Csn2H, and 392.4 for Csn3. These values are in the range of chitosanases previously studied by our group except for Csn3 which is considerably higher.

### Biochemical characterization

**Fig. 3:**
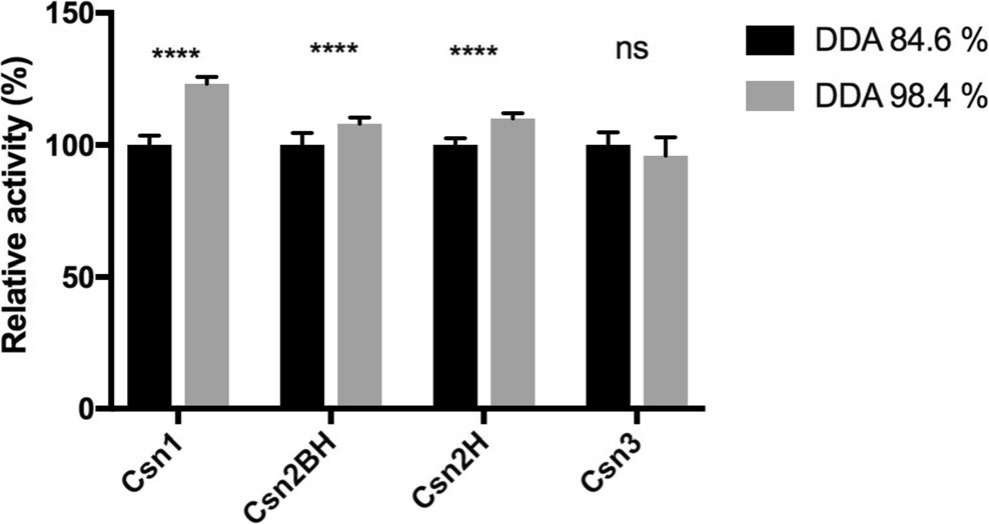
Activity of chitosanases against chitosans of two different DDAs. For each enzyme, activity on lower DDA chitosan was taken as a reference (100%). Results are averages of 12 (Csn1, Csn2H) or 18 (Csn2BH, Csn3) replicates. ****: activity on 98.4% chitosan significantly higher (*p* < 0.0001) than on 84.6% chitosan, ns: difference not statistically significant

It was shown previously that the specific activity of chitosanases can vary according to the DDA of chitosan and that a single mutation could alter the substrate preference profile (Shimosaka et al. 1995; Lacombe-Harvey et al. 2013). Here, we tested two chitosans with DDA of 84.6 and 98.4%, respectively (Fig. 3). Twelve replicas in two independent series of assays were performed. A statistical paired *t* test was used to estimate if a given enzyme is significantly more active on higher DDA chitosan than on lower DDA chitosan. For Csn1, Csn2BH, and Csn2H, the activity increased significantly with the increase of DDA, a profile similar to that observed for wild-type CsnN174 (Lacombe-Harvey et al. 2013) which shares high amino acid sequence similarity with Csn1 and Csn2. In contrast, Csn3 did not show any significant preference regarding DDA of chitosan substrate.

Measurement of kinetic parameters was performed with chitosan Sigma (Table 2). Substrate affinity, illustrated by *K*_m_, is roughly in the same range for Csn1, Csn2BH, and Csn2H but Csn3 has significantly higher *K*_m_ (and, consequently, lower substrate affinity) than the other three enzymes. Kinetic study showed also that the very high specific activity of Csn3 is essentially explained by its rapid turnover.

**Table 2:** Kinetic parameters of chitosanases

Enzyme	*K*_m_ (μg ml^−1^)	*K*_cat_ (min^−1^)
Csn1	21.9 ± 3.7	869
Csn2BH	15.8 ± 1.9	895
Csn2H	11.8 ± 1.6	660
Csn3	93.8 ± 12.5	28,388

Measurements were performed with chitosan from Sigma-Aldrich. Each concentration was assayed in six replicas

### Activity at low temperatures

**Fig. 4:**
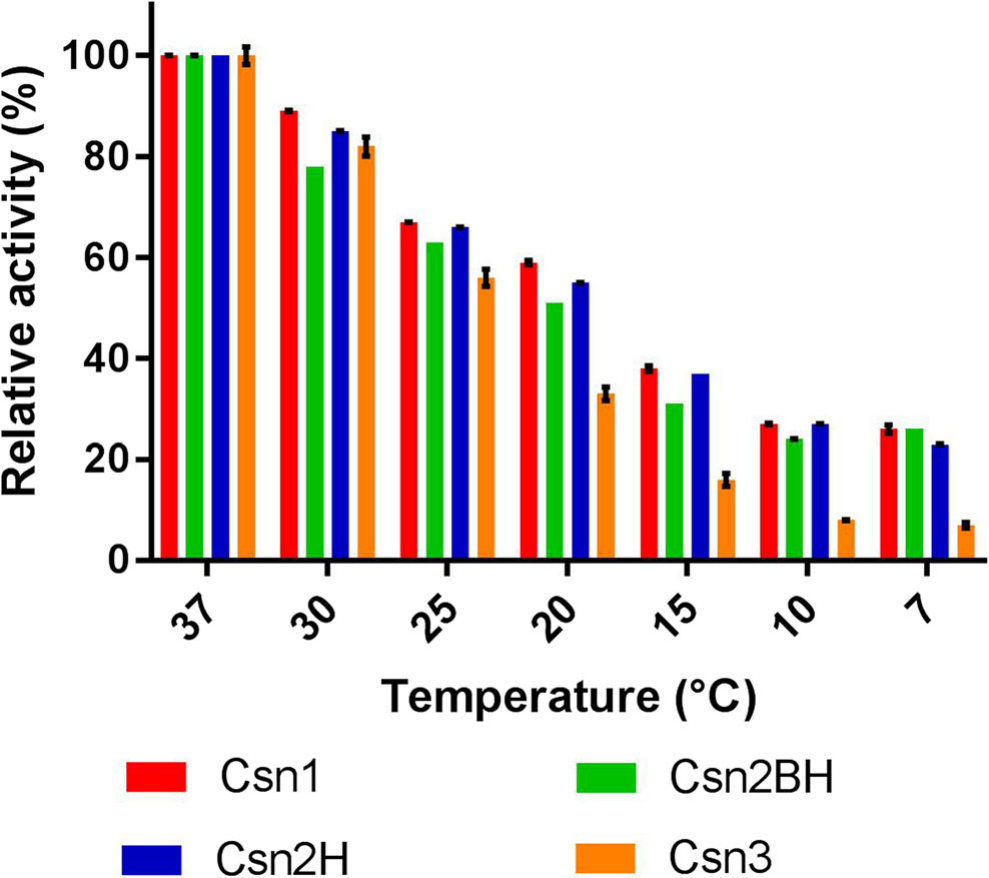
Activity of chitosanases at low temperatures with standard substrate. Activity at 37 °C was taken as a reference. Results are averages of six replicates

Enzymes studied in this work originate from a soil organism and are more often exposed to cold conditions than to temperatures of 30–40 °C typically used in laboratories. However, cold temperatures were rarely applied in mostly biotechnologically oriented chitosanase studies (Johnsen et al. 2010). To evaluate how the chitosanases perform in cold environment, the relative loss of activity (compared with standard conditions at 37 °C) was measured for a series of temperatures (Fig. 4). It resulted that Csn3 is much more sensitive to low temperatures than the other three enzymes. In the lowest studied range (7 to 10 °C), Csn3 kept only 7% of activity (compared to standard conditions at 37 °C), while the other three enzymes had 23 to 27% of residual activity.

### Activity on chitosan complexed with polyphosphoric and humic acids

*K*. *setae* is a soil organism and GH46 chitosanases are secreted outside the cell. In contrast with laboratory test conditions where chitosan is dissolved in buffer, enzymes in nature will have to act on undissolved chitosan or, perhaps more often, on chitosan being in interaction with a myriad of chemical compounds present in the soil environment. However, activity of chitosanases on complex substrates was rarely discussed in the literature (Sawaguchi et al. 2015). We then examined undissolved (i.e., freshly suspended) chitosan as substrate, as well as two compounds which are likely to form complexes with chitosan in soil.

Humic acid, universally present in soil, was shown to form complexes with chitosan (Wan Ngah and Musa 1998, Chen et al. 2011). The second tested compound is polyphosphate, a polymer commonly found in microbial cells (Achbergerová and Nahálka 2011). While synthesized and degraded intracellularly, it can be released in the environment as a result of cell lysis and form a complex with chitosan. Polyphosphate-chitosan complex is also of interest as it was used in many biotechnologically or agriculturally oriented studies (Frossard et al. 1994; Jobin et al. 2005).

**Fig. 5:**
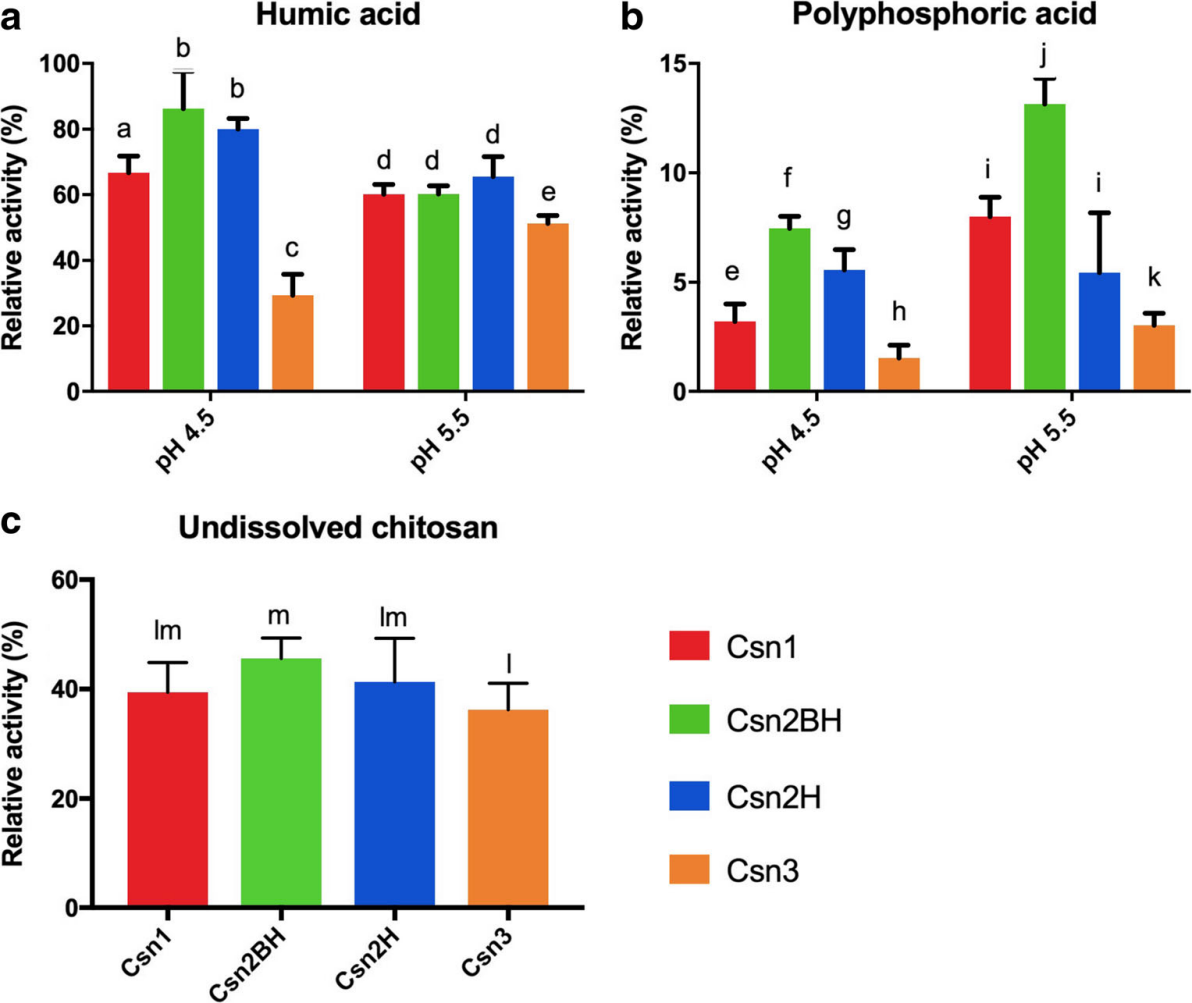
Activity of chitosanases on **a** chitosan complexed with humic acid. **b** chitosan complexed with polyphosphoric acid. **c** undissolved chitosan. Activity at a given pH with standard substrate (chitosan Sigma) was taken as a reference. Identical letters indicate that the activities do not differ significantly, as concluded from the Tukey’s multiple comparisons test. All assays were performed with six replicates

We tested several ratios of chitosan to polyphosphoric acid or humic acid at two pH values (4.5 and 5.5). Many of the tested conditions were either too severe for all enzymes (resulting in no measurable hydrolysis) or too mild (no differences among enzymes were observed and relative activity compared to that on sole chitosan was close to 100%). We present, for both complexes, the conditions that allowed for best differentiation among the relative activities of the four studied chitosanases. In such conditions (Fig. 5) (see also “Materials and Methods” for details), Csn3 showed significantly less relative activity than the other enzymes. In addition, Csn2 had better or equal performance compared with Csn1. Furthermore, Csn2BH had significantly better activity on chitosan complexed with polyphosphoric acid (Fig. 4b) than Csn2H. On the other hand, the relative activity on undissolved chitosan was similar for all polypeptides (Fig. 4c). These results underline again the biochemical diversity among the four chitosanases.

## Discussion

Heterogeneity in biochemical properties among GH46 chitosanases have been approached to some point in the literature. For instance, chitosanases from *Streptomyces* sp. N174 and *Bacillus circulans* MH-K1 differ by their preference to hydrolyze some linkages in chitosan. These differences have been explained by variations in tertiary structures (Saito et al. 1999). However, our work is focused on heterogeneity of GH46 chitosanases originating from the same one bacterial strain. Moreover, in most of the performed tests, at least one chitosanase stayed apart from the others. This is an indication that GH46 chitosanases are not biochemically homogenous despite their sequence similarity.

Csn1 was very similar to chitosanase CsnN174 from *Streptomyces* sp. N174 (actually shown to belong to the genus *Kitasatospora* by 16S RNA sequencing; Blanchard et al. 2001). The high degree of amino acid sequence identity among them is reflected by similar kinetic properties and pronounced preference for high-DDA substrates.

Csn2 was found in two forms. The shorter Csn2H comprising only the catalytic module was generated, possibly by proteolysis, from the full-length, bimodular Csn2BH which included a carbohydrate-binding module. Such proteolytic events targeting poorly structured segments, intercalating functional modules of proteins were observed in many instances (Watanabe et al. 1990; Gilkes et al. 1991). However, this is the first example of a well-documented cleavage of this kind in a GH46 chitosanase. In all our tests, the hydrolytic module itself, represented by Csn2H polypeptide, gave results similar to Csn1. But, due to the presence of CBM, the full-length Csn2BH chitosanase stayed apart from its shorter form in assays with chitosan complexed with polyphosphoric acid. We then suggest that a possible function of the carbohydrate-biding module consists in increasing the accessibility of the chitosanase to its substrate in situations where chitosan is “hidden” by a complexing partner.

Csn3 is a case apart. In almost all our experiments, Csn3 behaved differently from the other three enzymes. It has much higher specific activity (possibly due to its much more rapid turnover), no substrate preference regarding the DDA of chitosan, and it loses its activity more rapidly in the cold and is less performant against chitosan complexed with polyphosphoric or humic acid. According to two large alignments of GH46 protein sequences (Takasuka et al. 2014; Viens et al. 2015b), Csn3 belongs to a separate cluster (group B) mostly populated by chitosanases from Gram + bacilli with A + T-rich genomes. However, there are no signs that the *csn3* gene of *K. setae* could be laterally acquired from the *Bacillus* genus: DNA sequence analysis of *csn3* with FramePlot (Ishikawa and Hotta 1999) revealed that it has a high G + C content with maximization of G’s and C’s in the third position of codons, typical for actinobacteria but not for bacilli (data not shown).

**Fig. 6:**
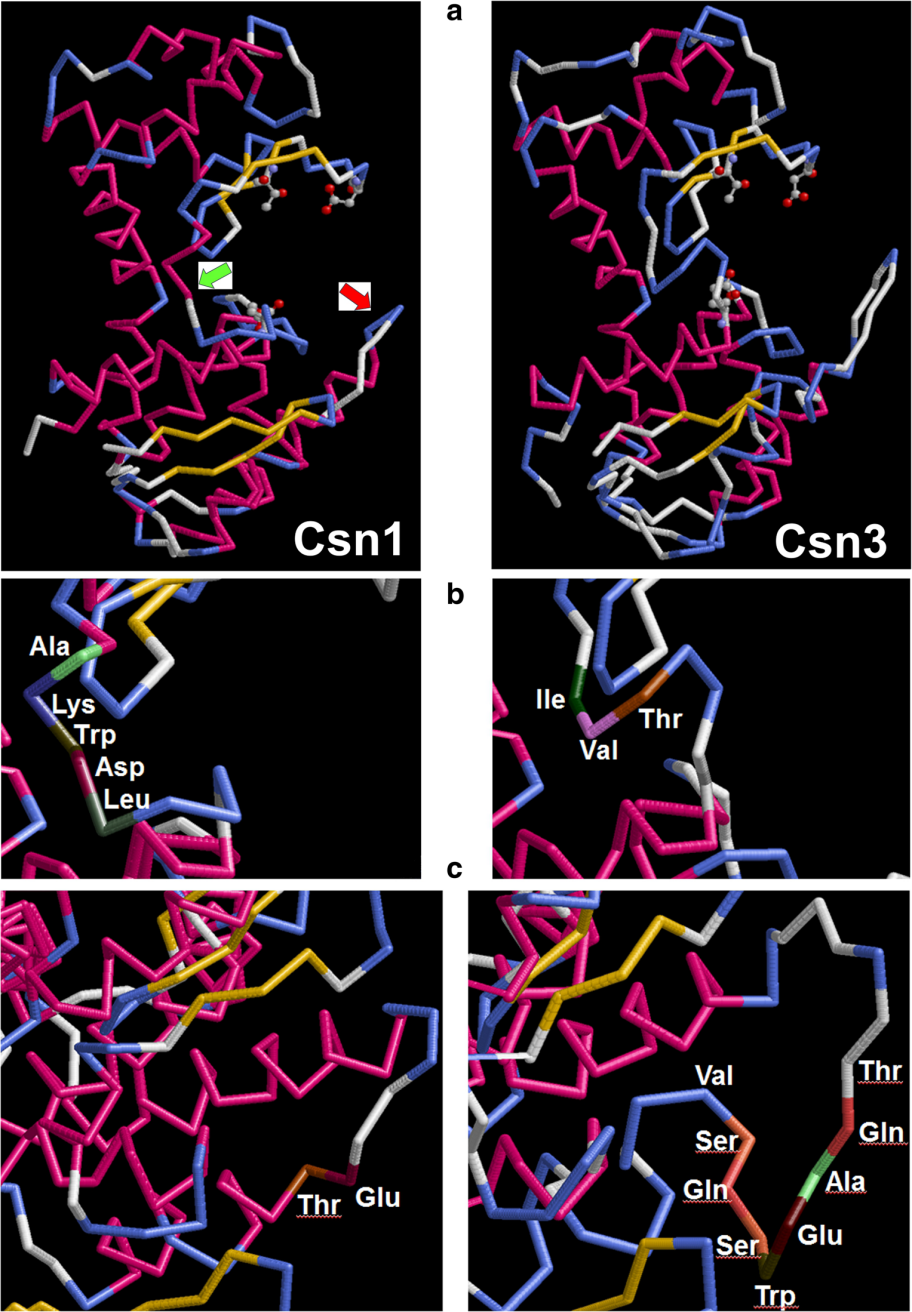
Major structural differences between chitosanases Csn1 and Csn3. **a** Overall structure models obtained with ESyPred3D (Lambert et al. 2002) using the 4OLT file from Protein Data Bank (Lyu et al. 2014) as template, showing the minor (upper) lobe and the major (lower) lobe. Three residues directly involved in catalysis (Glu, Asp, Thr) are represented as balls and sticks. Green and red arrows indicate the segments detailed in sections **b** and **c**, respectively. **b** comparison of hinge segments between the major and the minor lobe. **c** comparison between large loops in the major lobe. Graphics executed with the RasMol program (Sayle and Milner-White 1995)

The comparison of 3D models of Csn1 and Csn3 chitosanases (Fig. 6a) highlights two major structural differences. First, the hinge segment linking the minor lobe and the major lobe close to the N-terminus is shorter by two residues in Csn3 (Fig. 6b). The general acid catalytic residue (a glutamate) is localized in the proximity of this hinge segment (Marcotte et al. 1996; Lyu et al. 2014). Second, a large loop closing the substrate-binding cleft in the major lobe is longer by seven residues (Fig. 6c). An important arginine residue is localized in the immediate vicinity of this loop. Such structural differences could influence the function of these crucial residues. Also, a shorter hinge segment could influence the rotation of the minor lobe relative to the major lobe occurring during the process of substrate binding (Lyu et al. 2015).

Other differences among Csn3 and Csn1 as well as other deeply studied chitosanases from group A have possibly predictable consequences. Lyu et al. (2014, 2015) analyzed in detail the catalytic and substrate-binding mechanisms of chitosanase OU01. Based on their work and on the large sequence alignment published previously (Viens et al. 2015b), we extracted a list of residues important for catalytic mechanism and substrate binding, as well as those contributing to the electronegativity of the substrate-binding cleft and the stability of inter-lobe interactions. We present (Table 3) the corresponding residues in CsnN174, Csn1, Csn2, Csn3, and the chitosanase from *Bacillus subtilis* (Rivas et al. 2000).

**Table 3:** Correspondence of functional residues among chitosanases

Function	group A				group B	
	OU01 chitosanase	CsnN174	Csn1	Csn2	Csn3	B.subt.
Catalysis						
General acid	Glu25 (E25A = ▼)	Glu	Glu	Glu	Glu	Glu
General base	Asp43 (D43A = ▼)	Asp	Asp	Asp	Asp	Asp
Water orientation	Thr48	Thr	Thr	Thr	Thr	Thr
Substrate binding						
− 3, H-bond (NH_2_)	His153	His	His	His	His	His
− 3, H-bond (OH)	Pro155	Pro	Pro	Pro	Asn	Asp
− 3, H-bond (OH)	Thr58	Thr	Thr	Thr	Asp	Thr
− 2, H-bonds (OH)	Arg45	Arg	Arg	Arg	Arg	Arg
− 2, H-bond (NH_2_)	Asp60 (D60E = ▼)	Asp	Asp	Asp	Asp	Asp
− 2, H-bond (NH_2_)	Gly53	Gly	Gly	Gly	Gly	Gly
− 2, H-bond (NH_2_)	Ile52 (through H_2_O)	Ile	Ile	Ile	Ala	Ala
− 2, H-bond (NH_2_)	Val151 (through H_2_O)	Val	Val	Val	Val	Ile
− 2, H-bond (OH)	Asp60	Asp	Asp	Asp	Asp	Asp
− 2, H-bond (OH)	Thr58	Thr	Thr	Thr	Asp	Thr
− 1, H-bond (OH)	Gly53	Gly	Gly	Gly	Gly	Gly
− 1, H-bond (OH)	Ile52	Ile	Ile	Ile	Ala	Ala
− 1, H-bond (OH)	Ile52 (through H_2_O)	Ile	Ile	Ile	Ala	Ala
− 1, H-bond (OH)	Val151 (through H_2_O)	Val	Val	Val	Val	Ile
− 1, H-bond (NH_2_)	His203 (H203A = ▼)	His	His	His	Ser	His
− 1, H-bond (OH)	His203 (H203A = ▼)	His	His	His	Ser	His
+ 1, H-bond (OH)	Ala202	Ala	Ala	Ala	Asn	Asn
+ 1, H-bond (NH_2_)	Tyr37	Tyr	Tyr	Tyr	Tyr	Tyr
+ 2, H-bond (OH)	Ser27	Ser	Ser	Ser	Gly	Gly
+ 3, H-bond (OH)	Ser27	Ser	Ser	Ser	Gly	Gly
+ 3, H-bond (NH_2_)	Asp235	Asp	Asp	Glu	Ala	Tyr
Cleft electronegativity						
	Glu39	Glu	Glu	Glu	Glu	Glu
	Asp40	Asp	Asp	Asp	Asn	Arg
	Asp60	Asp	Asp	Asp	Asp	Asp
	Glu63 (E63A = ▲)	Glu	Glu	Asp	Lys	Glu
	Glu120 (E120A = ▲)	Glu	Glu	Glu	Gln	Val
	Glu200	Glu	Glu	Glu	Pro	Pro
	Asp235	Asp	Asp	Glu	Ala	Tyr
Inter-lobe stability	Arg123	Arg	Arg	Arg	Glu	His
	Glu120 (E120A = ▲)	Glu	Glu	Glu	Gln	Val

Numbering of residues and their function assigned for OU01 chitosanase as presented by Lyu et al. (2014, 2015). Corresponding residues in other chitosanases have been extracted from the structure-guided alignment in Viens et al. (2015b). In the substrate-binding section, the number of the subsite is indicated followed by the nature of interaction between amino acid and substrate. (= ▼) loss of activity after mutation into alanine; (= ▲) gain of activity after mutation into alanine as observed by Lyu et al. (2014, 2015)

Data in Table 3 allow for several conclusions. While members of group A chitosanases are remarkably homogenous in their functional residues (with just a few minor substitutions for Csn2), Csn3 is quite different, having more similarity to *B*. *subtilis* chitosanase. However, residues directly involved in catalysis are strictly conserved in all five enzymes. Inside the set of residues involved in substrate binding, several substitutions are observed in Csn3; what could explain the differences in *K*_*m*_ among Csn3 and Csn1 or Csn2H? Nevertheless, the most drastic substitutions are observed for residues creating the electronegativity of the substrate-binding cleft or those involved in the inter-lobe stability. In Csn3 (and *B*. *subtilis* chitosanase), the substrate-binding cleft appears to be much less electronegative, including only two acidic residues compared with seven in group A enzymes. Lyu et al. (2015) mutated two glutamates from this set into alanine and the resulting enzymes gained enhanced specific activity. Thus, the decrease of electronegativity of the substrate-binding cleft can explain, at least partly, the higher specific activity (and turnover number) of Csn3 compared with Csn1 and Csn2H.

Finally, it was postulated that two interacting residues, Glu120 and Arg123 in OU01 chitosanase contribute to inter-lobe stability (Lyu et al. 2015). These residues are substituted by Gln and Glu, respectively in Csn3 suggesting a weaker interaction resulting in increased inter-lobe flexibility. The E120A mutation introduced by Lyu et al. (2015) aiming to disrupt this interaction resulted in an enzyme with enhanced activity. Therefore, the increased inter-lobe flexibility provides a further explanation of the higher specific activity of Csn3. Clearly, group B chitosanases would merit more investigations of their structure-function relationships.

Members of the genus *Kitasatospora* were identified among the most abundant chitosan-degrading microorganisms at early stages of chitosan degradation in soil (Sawaguchi et al. 2015). The biochemical diversity of chitosanases such as observed in *K*. *setae* KM-6054 could contribute to such successful propagation. It remains to be investigated how such diversity could be exploited in biotechnological processes.
